# The Pelvic Support Osteotomy: A Useful Therapeutic Alternative for Chronically Unstable Hips in Children and Adolescents

**DOI:** 10.3390/children12101330

**Published:** 2025-10-03

**Authors:** César Salcedo Cánovas, Javier Martínez Ros, José Molina González, Juan Pedro García Paños, Sarah Toledo García, María José Ros Nicolás

**Affiliations:** Pediatric Orthopedics Unit, Virgen de la Arrixaca University Hospital (CSUR—National Reference Center for Pediatric Orthopedics, ERN BOND—European Reference Network on Rare Bone Disorders), Pediatric Orthopedics and Bone Reconstruction Research Group, IMIB-Arrixaca (Murcian Institute of Biosanitary Research), 30120 Murcia, Spain; drjmtnezros@gmail.com (J.M.R.); jmolinagonz@yahoo.com (J.M.G.); juampeboss@gmail.com (J.P.G.P.); sarah.toledo4@gmail.com (S.T.G.); rosn.mariaj@yahoo.com (M.J.R.N.)

**Keywords:** osteotomy, Ilizarov, hip, lengthening, fixator, dysplasia

## Abstract

**Highlights:**

**What are the main findings?**

- Pelvic support osteotomy combined with femoral lengthening effectively corrects leg length discrepancy and improves gait in children and adolescents with severely damaged hips.
- The procedure achieves significant functional improvement, with a marked reduction in Trendelenburg sign and restoration of limb length.

**What is the implication of the main finding?**

- Pelvic support osteotomy is a safe and viable alternative to arthrodesis or total hip arthroplasty in young patients with complex hip deformities.
- This technique should be considered in treatment planning for pediatric and adolescent patients where conventional options are limited.

**Abstract:**

Background/Objectives: The sequelae from conditions affecting the proximal femur may cause instability, pain, leg length discrepancies and abnormal gait. Treatment options include arthrodesis and total hip arthroplasty, but both alternatives have limitations in young patients with severe deformities. Pelvic support osteotomy constitutes a viable option in these cases. The present study analyses the effectiveness and safety of the procedure. Methods: This was a retrospective observational study on patients with an unstable or stiff hip treated with a pelvic support osteotomy. Both the results obtained and the complications that occurred were subjected to a statistical analysis. In addition, a narrative literature review was carried out to elucidate the biomechanical rationale and the results of the technique. Results: This study included a total of 12 patients (8 male and 4 female) with a mean age of 13 years (range: 0–19). All cases were unilateral and the mean follow-up time was 6.9 years (range: 1–10). Preoperative leg length discrepancy was 8 cm (range: 5–10), and all patients presented with a marked Trendelenburg sign. The mean leg lengthening achieved was 8 cm (range: 8–10), following a mean external fixation time of 263 days (range: 180–360), which entails an external fixation index of 32.5 days per centimeter lengthened (range: 25–37). Mean leg length discrepancy fell to 0.9 cm (range: 0–3) and the Trendelenburg sign improved following treatment: it disappeared in three patients (25%), it became mild in seven (58%), and it improved to moderate in two (17%). Eight patients (66%) experienced some sort of complication over the course of treatment. Conclusions: Pelvic support osteotomies, combined with femoral lengthening, are a safe and effective option for managing severely damaged hips in children and adolescents.

## 1. Introduction

Stability is a basic requirement for the proper functioning of any joint, the hip being no exception. Patients with sequelae from conditions affecting the proximal femur typically present with instability, as well as bone loss and proximal migration of the femoral bone, often accompanied by complications such as pain, leg length discrepancy, reduced range of motion and abnormal gait, which severely impact their quality of life [1]. The origin of the problem is typically multifactorial with congenital deformities, developmental dislocations, traumatic injuries, septic or tuberculous arthritis, avascular necrosis and poliomyelitis amongst the most likely culprits [1,2,3,4,5]. The issue is of particular concern as the problem arises during the first years of life and, although usually characterized by a painless onset, it subsequently gives rise to adaptive deformities, which, if not corrected early, form and develop progressively, often leading to difficult-to-resolve situations, including joint instability; excessive muscle, tendon and ligament strain; ventral pelvis rotation, lumbar hyperlordosis, shortening of the abductor lever arm, gluteus medius insufficiency, leg length discrepancy and Trendelenburg gait [2].

The main goal in treating an unstable hip is to reduce pain, improve joint range of motion, and equalize limb length [4,6]. However, although surgeons can choose from multiple treatment options, there is still some controversy as to which of them is the most appropriate one. Arthrodesis results in effective pain relief, but has been associated with decreased range of motion, pain in the lumbar region and in the contralateral hip and knee, and limping. Total hip arthroplasty (THA) achieves a good restoration of biomechanical function and is in fact the gold standard for patients with untreated high-riding hip dislocation [7]. Nonetheless, in the most extreme cases, anatomical alterations, poor bone quality and the high activity level of patients—often children or adolescents—have been shown to lead to a very high risk of prosthetic failure in the short term [7,8,9].

Pelvic support osteotomies (PSO) are another option for managing patients with severe instability. The first few reports on the technique date back to Bouvier in 1838. Later on, Kirmission suggested that femoral osteotomies could be useful in correcting the adduction contractures observed in patients with irreducible dislocations. The technique was refined by Von Bayer, Lorenz and Schanz, who proposed that increasing the contact area between the pelvis and the femur while reducing at the same time the limb’s adduction range could improve the patients’ Trendelenburg gait in cases presenting with irreparable damage to the hip’s original anatomy [10]. Various solutions have been described to achieve this goal. Of particular interest is the technique proposed by Ilizarov [11,12], who designed a proximal osteotomy combining varus correction, extension and derotation of the limb, creating a weightbearing surface between the proximal femur and the distal pelvis. A diaphyseal osteotomy was also added to counteract the valgus forces resulting from the proximal osteotomy and to lengthen the limb in order to compensate for potential leg length discrepancies.

Successful PSO have been shown to relieve pain, reduce limping, equalize limb length and result in a more effective restoration of gait [3]. Unlike other treatment alternatives, which have provided relatively unsatisfactory or unpredictable results, PSO could be considered a valid option for the management of hip instabilities in adolescents and young adults [13].

The purpose of this study was to analyze the effectiveness and safety of Ilizarov’s PSO as a therapeutic option in pediatric patients with sequelae from various conditions affecting the hip. In addition, a narrative literature review was conducted to gain a better understanding of the state of the art of the technique.

## 2. Materials and Methods

This was a retrospective observational study on patients diagnosed with an unstable and/or stiff hip treated with PSO at the Virgen de la Arrixaca University Hospital between 2011 and 2021. This study was approved by the research evaluation committee of Area I of the Murcia Health Service on 21 March 2022. All patients and their legal guardians provided their informed consent to participate in this study after being informed about its nature, objectives and implications.

Patients who did not give their consent to participate, as well as those presenting with active infections at the time of surgery, those bearing a hip prosthesis (or due to be implanted with one in the short term), and those for whom information about their medical record or the imaging tests conducted over the course of treatment had been lost, were excluded from this study.

Patients were analyzed before and after treatment. Demographic variables (age and sex), as well as variables related with the condition (etiology, leg length discrepancy, joint range of motion, and Trendelenburg gait severity [14]); the treatment (rate and degree of limb lengthening, duration of external fixation and external fixation index), and the potential complications (as evaluated by Paley’s classification) were duly recorded.

A descriptive analysis of the data was performed, using measures of central tendency and dispersion. Inter-group comparisons were made with either parametric or non-parametric means difference tests, depending on the normality of the samples. Qualitative variables were analyzed by means of Pearson’s chi squared test or the Fischer Exact Test, depending on the magnitude of expected values. Effect size was calculated using Cohen’s d. Statistical significance was in all cases set at a p value of 0.05. The data was analyzed using R software (R Development Core Team), v. 4.1.3.

A narrative literature review was carried out of the previously published series on the subject and of various analyses on the biomechanical and clinical aspects of PSO.

### Surgical Technique

Although the surgical technique has been extensively described by other authors [3,11,12,13], a series of controversial aspects will be highlighted here in order to justify the treatment alternative selected in the present case series.

**Figure 1:**
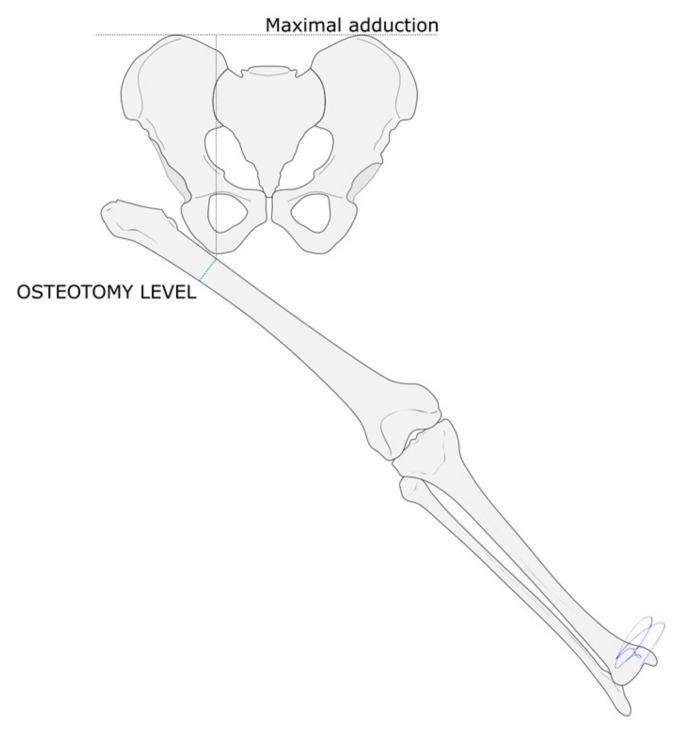
The level of the proximal osteotomy is set at the intersection between a vertical line extending distally from the ischial tuberosity and the anatomical axis of the femur, with the latter in full adduction.

**Figure 2:**
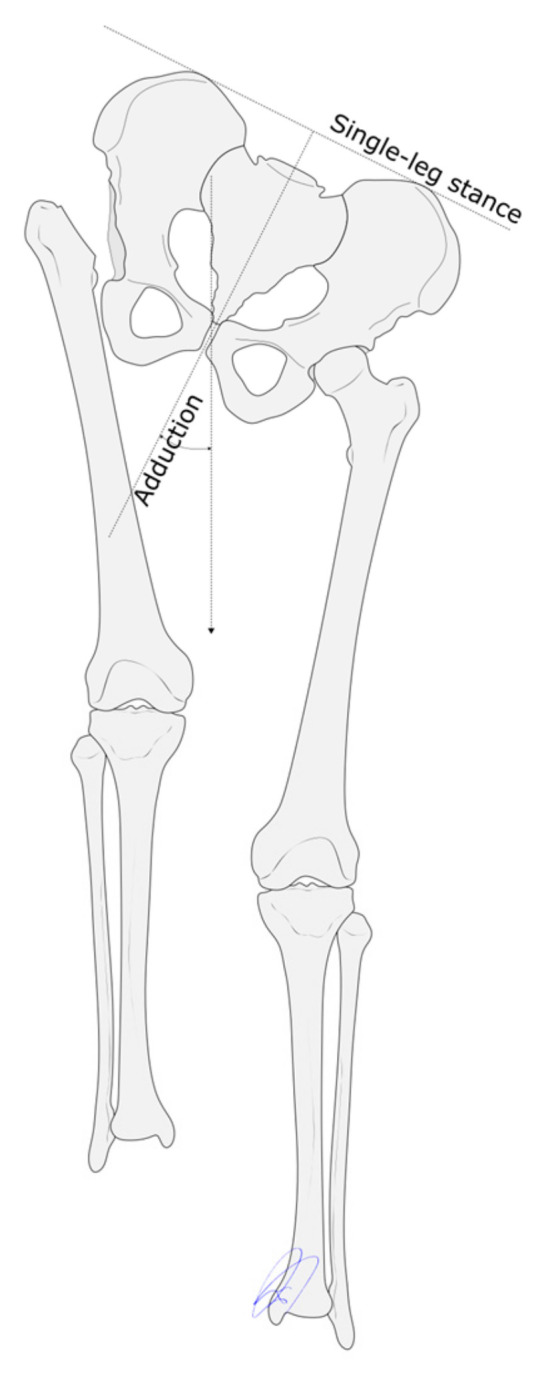
The angle resulting from the proximal osteotomy is calculated by adding 15 degrees to the adduction exhibited by the patient during single-leg loading.

Given the need that the hip joint be reasonably mobile for the pelvic osteotomy to be successful [10,15], a femoral head resection was performed in cases where this requirement was not met. The optimal level at which the proximal osteotomy must be carried out remains a matter of controversy [16,17,18]. In the present series it was decided to make the osteotomy at the intersection between a vertical line extending distally from the ischial tuberosity and the anatomical axis of the femur, with the latter in full adduction [7,13] (Figure 1). In addition, in order to compensate for the stresses generated by the abductor muscles, the proximal osteotomy had to produce a valgus correction of 15 degrees, capable of increasing the patients’ adduction during single-leg loading [13] (Figure 2). The angular correction achieved by the osteotomy should resolve the patients’ flexion contracture and correct their lumbar hyperlordosis. However, following the proximal osteotomy, the femur is typically in full adduction and externally rotated. This makes it necessary to apply a compensatory internal rotation maneuver at the site of the proximal femoral osteotomy [13]. The goal of the distal osteotomy is to give rise to a well-aligned limb by means of a varus correction of the distal femur and a bone lengthening procedure to resolve the patient’s leg length discrepancy.

**Figure 3:**
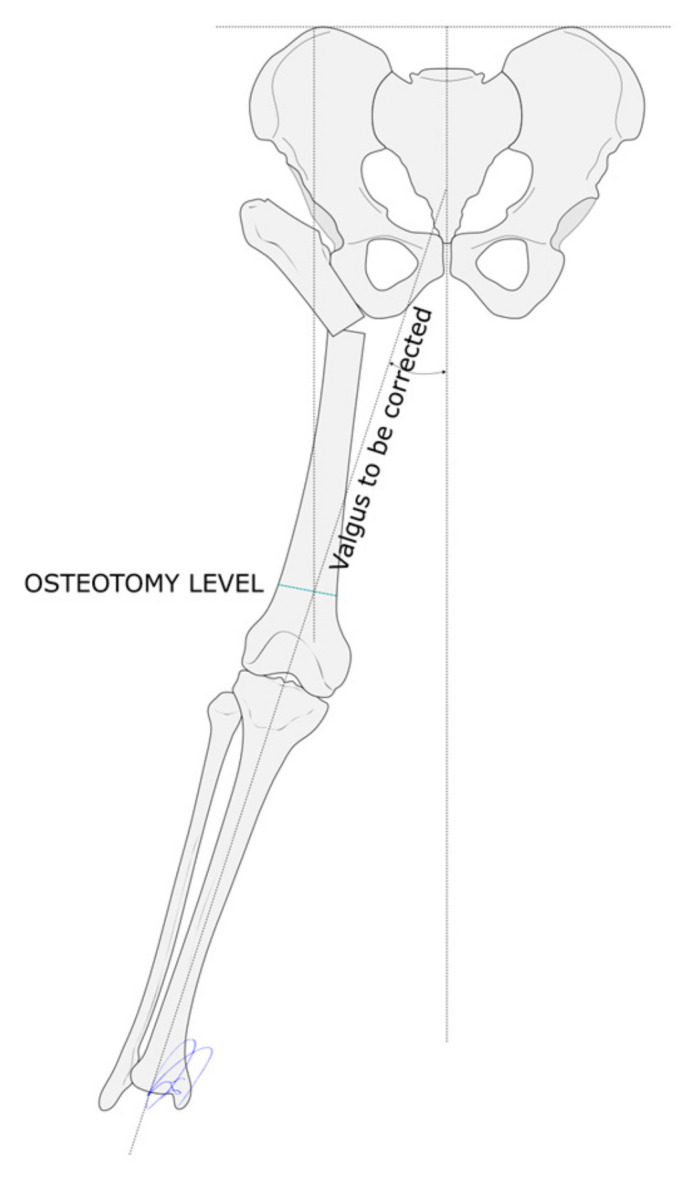
The distal osteotomy must be performed at the intersection between a straight line perpendicular to the Hilgenreiner line that runs through the proximal osteotomy site and another line that follows the mechanical tibial axis.

**Figure 4:**
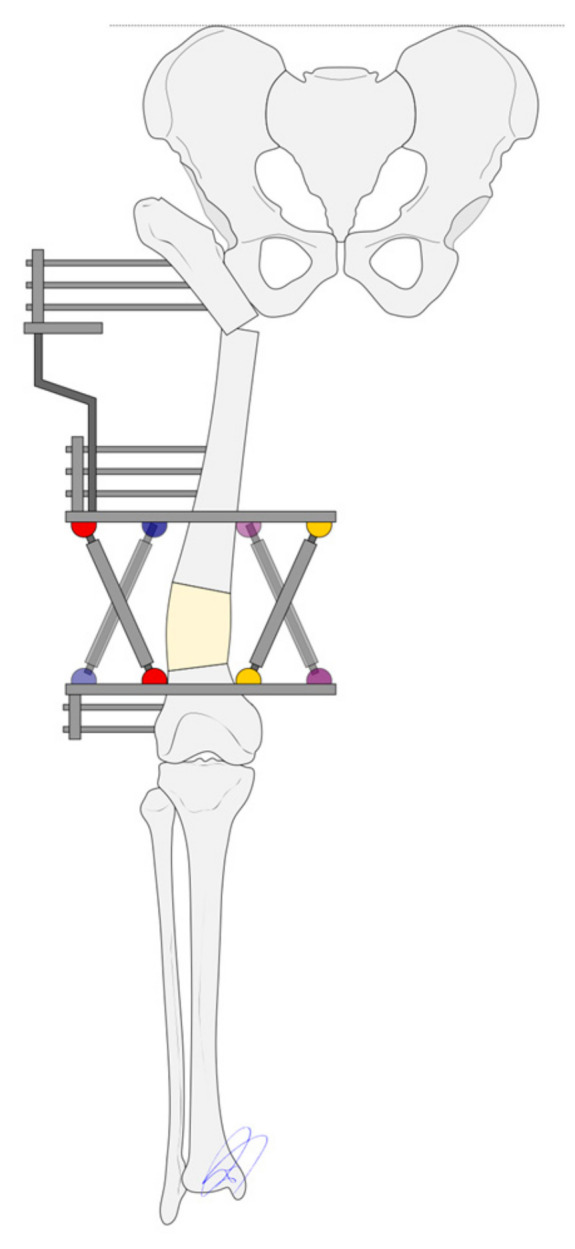
An external fixator must be used to correct the resulting varus deformity and lengthen the limb.

The limb is considered to be fully aligned when a straight line that runs perpendicularly to the Hilgenreiner line and crosses the proximal osteotomy site passes through the center of the knee and ankle joints. This means that the distal femoral osteotomy must be performed at the intersection between the proximal and the distal mechanical axes. The proximal axis can be determined by drawing a straight line perpendicular to the Hilgenreiner line that runs through the proximal osteotomy; the distal axis is a straight line that passes through the center of the ankle and knee joints (Figure 3). The femoral varus deformity must be corrected to the extent that it gives rise to a well-aligned limb. The amount of lengthening required is determined based on preoperatively-performed lower limb teleradiographs (Figure 4).

## 3. Results

This study included a total of 12 patients (8 male and 4 female), with a mean age of 13 ± 3.2 years (range: 9–19). All patients were able to ambulate independently, although some required the use of walking aids (crutches), either continuously or intermittently. The different etiologies and the patients’ baseline characteristics are summarized in Table 1. Severe avascular necrosis was documented in 9 (75%) patients; dislocation in 3 (25%) and joint stiffness in 8 (67%). One of the patients presented with coxa vara (8%) and another coxa valga (8%). One case of genu valgum (8%) completed the roster of anatomical defects recorded in the sample. All cases were unilateral, the right hip being the most affected one (75% [n = 9] of patients). Mean follow-up was 6.9 ± 3.0 years.

Preoperative leg length discrepancy was 8 ± 1.7 cm (range: 5–10). Mean hip extension was 7.5 ± 13.6 degrees, mean hip flexion 96.7 ± 15.6 degrees, internal rotation 9.2 ± 10.0 degrees, external rotation 10 ± 13.5 degrees, abduction 18.3 ± 11.1 degrees and adduction 12.5 ± 6.2 degrees. All patients presented with a severe Trendelenburg sign prior to being treated (Table 2). 

**Figure 5:**
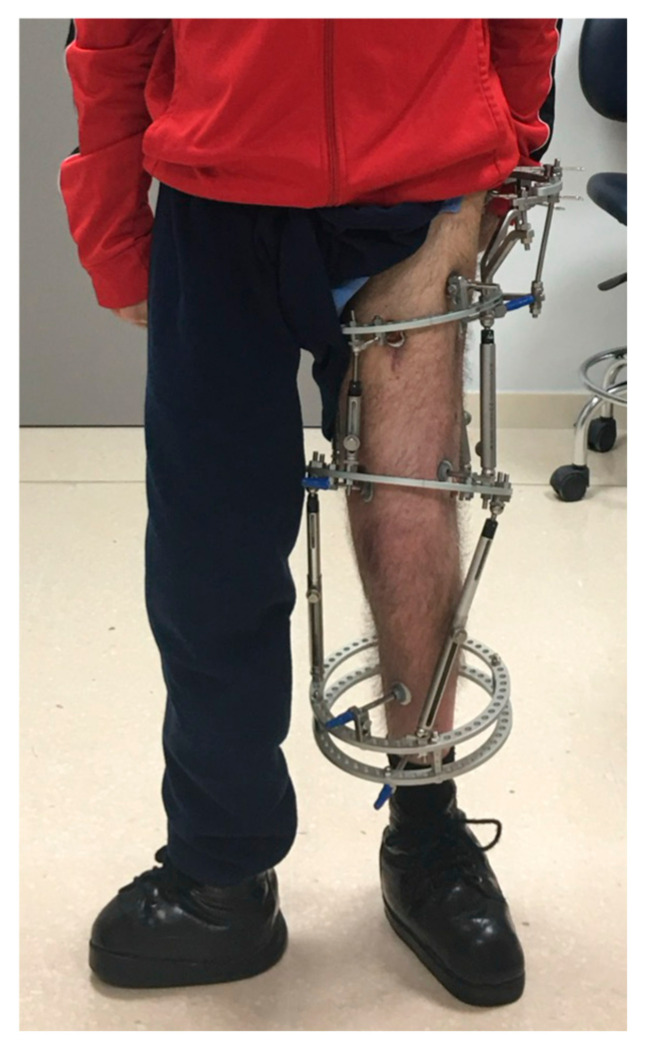
One of the patients in the series, bearing a TrueLok circular external fixator (Orthofix Srl, Verona, Italy).

**Figure 6:**
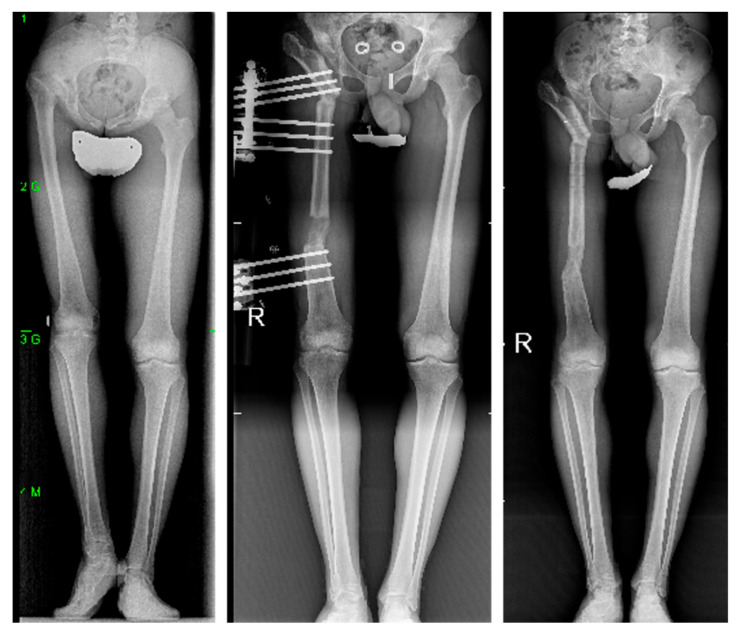
Radiographic evolution of a 15-year-old male with developmental dysplasia of the hip, multiple previous surgeries, a painful stiff hip, a 6 cm leg length discrepancy and severe Trendelenburg gait, treated with an LRS Advanced monolateral external fixator (Orthofix Srl, Verona, Italy).

The most frequently used external fixator was the LRS Advanced monolateral fixator (Orthofix Srl, Verona, Italy), followed by the TrueLok circular fixator (Orthofix Srl, Verona, Italy) and the Monotube Triax monolateral fixator (Stryker Corp, Kalamazoo, MI, USA) (Figure 5 and Figure 6). The mean limb length achieved was 8.1 ± 1.6 cm, at a mean elongation rate of 0.96 ± 0.1 mm daily. Mean distraction time was 84.2 ± 18.6 days and the mean duration of external fixation stood at 263 ± 59.6 days, which entails a mean external fixation index of 32.5 ± 3.4 days per centimeter lengthened.

The patients’ final outcomes demonstrate a statistically significant and clinically meaningful improvement across all analyzed parameters. Mean leg length discrepancy fell to 0.9 ± 1.1 cm (*p* = 0.002, r = 0.887), mean hip extension rose to 24.2 ± 5.2 degrees (*p* = 0.006, r = 0.796), mean flexion increased to 128 ± 8.7 degrees (*p* = 0.002, r = 0.889), internal rotation improved to 24.2 ± 5.2 degrees (*p* = 0.004, r = 0.860), external rotation climbed to 38.3 ± 3.4 degrees (*p* = 0.003, r = 0.886), the abduction angle escalated to 37.5 ± 4.5 degrees (*p* = 0.002, r = 0.897) and the adduction angle went up to 22.5 ± 4.5 degrees (*p* = 0.003, r = 0.882) (Table 3).

The patients’ Trendelenburg sign improved significantly (*p* = 0.002) following treatment, from a situation where all patients exhibited severe gait abnormalities to one where 25% of them (3 patients) presented with no Trendelenburg sign, 58% (7 patients) demonstrated a slight gait dysfunction and 17% (2 patients) showed moderate Trendelenburg gait. Sixty-six percent of the sample (8 patients) developed some kind of complication over the course of treatment (Table 4). The most common complication was pin tract infection (n = 3), followed by delayed healing (n = 2), fracture of the regenerate (n = 2), joint contractures (n = 2) and sciatic neuritis (1 case). Overall, 70% of the complications were classified as problems, 10% as obstacles and 20% resulted in sequelae.

Table 5 summarizes the main results of the articles found in the literature dealing with the PSO technique. These data are included as context for the results from the present series.

## 4. Discussion

The PSO technique has over the years generated no small amount of controversy. This is probably so because the realities facing pediatric orthopedists differ greatly from those faced by surgeons treating adult patients. Children are indeed not small adults and the solutions that may solve the problems of children are not necessarily those best suited for adults. In our series of 12 patients, PSO combined with distal femoral lengthening provided significant improvements in pain, leg length discrepancy, and overall function. All patients were able to ambulate independently, and despite a relatively high rate of minor complications, no major adverse events occurred. These findings support the role of PSO as a valuable salvage procedure in this challenging patient population.

Nevertheless, it should be pointed out at the outset that the authors are not proposing that PSO should be the treatment of choice for all children with hip abnormalities. In fact, its role in clinical practice is very limited [10] and it is not indicated in patients amenable to THA, or even arthrodesis [2,3,10]. Nevertheless, the technique is a good alternative in extreme cases, such as in very young patients presenting with a severe dislocation associated with Trendelenburg gait and lumbar hyperlordosis. Given their low weight and great flexibility, these subjects may present with no pain and may be more tolerant of deformities. However, if the latter are not corrected early, they are likely to worsen and become chronic [2,3,13]. Moreover, protheses are not always a viable option due to these patients’ short stature and to the short survival of implants in this patient group [7,8,9]. At the same time, it should be remembered that PSO is contraindicated in the presence of an active infection, in non-ambulatory patients, in patients with a stiff hip or in those unable to tolerate long external fixation periods [2,3,10,19]. Nevertheless, and although stiffness is usually regarded as a contraindication for this technique, in our series, we also applied it in stiff hips by performing femoral head resection. In these cases, we combined the main approach with an anterior approach to create an interposition arthroplasty. Our experience shows that this strategy provides good outcomes despite the initial stiffness.

There is no universally accepted option for the management of chronically unstable hips. Although arthrodesis typically results in a stable and painless joint, it reduces hip range of motion and negatively impacts the lumbar spine, in addition to increasing leg length discrepancy [22]. THA has been shown to successfully restore joint biomechanics, relieving pain and providing a good range of motion [4], but fitting a prosthesis into a deformed hip is technically challenging and has been associated with a high rate of complications [2,4,8,9,18,21,27]. This means that the use of THA in young patients is at best controversial [28]. Although it is true that the most recent literature has reported more encouraging survival rates than those obtained in studies conducted prior to 1995 [29,30,31], muscle contractures, deformities, poor bone quality, narrow medullary canals and the high activity level of young patients tend to result in high prosthetic failure rates [7,9,21,27]. Even in the case of successful arthroplasties, the limited lifespan of joint replacements means that young individuals must undergo several revision procedures during their lifetime, the complexity of such procedures being typically higher in patients with pre-existing anatomical deficiencies [4]. All the foregoing has led some authors to reject THA as the treatment of choice in young individuals [8,9], while others advocate conservative treatment methods, claiming that surgery ought to be postponed until the patient is older, some of them even suggesting the age of 40 years as a threshold for undergoing the procedure [18].

In contrast with the above, the revision rates found in our PSO literature review were practically negligible and, in the case of this series, it was zero percent. Some authors have considered PSO a procedure with lifelong results [5], with some patients operated over 50 years ago still maintaining their gains in terms of hip range of motion, ambulation and pain relief [20]. However, it should be pointed out that this is not the case in younger patients, who face a high risk of recurrence due to their high bone remodeling potential. In many of them, correction of the proximal femoral angle may be lost over time, making a revision surgery necessary [7,13]. Loss of correction among patients aged between 9 and 17 years has been found to range between 3 and 13 degrees [32], which has prompted some authors to contraindicate PSO in individuals below the age of 12 years [2,3,6,28], and others to recommend deferring the procedure until the age of 15 years [7,32]. Based on our belief that the functional improvement gained following PSO, combined with the avoidance of the compensatory deformities likely to arise in its absence, clearly outweighs the risk of having to perform a potential revision surgery, we do not consider that any age limits should be established.

One of the most immediate effects of PSO is an improvement in gait performance, with a notable reduction and even disappearance of the Trendelenburg sign, which is associated with disturbances in the adjacent joints and with the presence of pain and fatigue, particularly at the end of the day [3,5,13]. Our literature review showed that the Trendelenburg sign disappears in up to 67% of patients treated with PSO, which can be explained by several factors. Lateralization of the greater trochanter tightens the gluteus medius, increasing its efficiency and limiting the descent of the pelvis during gait [3]. This effect is supplemented by the medial support provided by the femur, which avoids adduction of the limb and prevents pelvic drop [3]. No other treatment method—except for arthrodesis—has been shown to successfully address this issue [13]. It must be said, however, that older patients do exhibit a more modest reduction in their Trendelenburg sign [21], with some of them experiencing relapses attributable to a potential disuse weakness of their abductor muscles or to the development of a pressure atrophy of the tissues interposed between the angulated proximal femur and the lateral wall of the pelvis, which allows for a certain degree of adduction and the return of a positive Trendelenburg sign [15]. As mentioned in the section dedicated to the surgical technique above, some authors recommend hypercorrecting the angle resulting from the osteotomy of the proximal femur, yet others consider that such a hypercorrection could make the results less predictable. In any event, it should be stated that although patients experience a clear improvement following PSO, long walks still make them feel exceedingly fatigued [5]. In our series, the increase in range of motion was related to the femoral head resection and interposition arthroplasty, which prevent the limitation caused by joint blockage and allow for an immediate gain in mobility.

An additional advantage of PSO has to do with cosmetics. The angulation resulting from the osteotomy of the proximal femur is masked by the soft tissue mass surrounding the proximal aspect of the femur; in addition, this mass tends to shift laterally conferring the area with a normal appearance. Moreover, the functional abduction obtained on the operated side helps with perineal hygiene and enhances sexual function in women.

There has been a certain measure of debate in the literature as to whether PSO is capable of eradicating pain. Although the majority of articles reviewed report significant levels of pain relief, some authors contraindicate the technique in cases where pain is the patient’s main complaint, arguing that the pain relief resulting from the procedure is not always predictable [10]. Conversely, other authors have argued that this is precisely the aspect where the patients’ improvement is most remarkable [28]. In our case, although tissue transfixion and limb lengthening undeniably result in a certain measure of chronic dull pain, which can be controlled with analgesics, the treatment has been shown to be effective in eradicating the pain experienced by some of the patients in the series.

As regards complications, this series confirms the findings of previous reports. Indeed, the majority of complications were related to the external fixator (pin tract infections) and the limb lengthening procedure (fractures of the regenerate and healing problems), which are also frequently reported in other published series. The only two obstacles were resolved through minor arthrolysis procedures without leaving long-term sequelae. Overall, these findings suggest that, although complications may occur, they are generally minor and do not compromise the positive clinical outcomes achieved with pelvic support osteotomy combined with distal femoral lengthening.

The mean duration of external fixation in the literature is 6.9 months (8.8 months in our series). This long duration has prompted several authors to contraindicate PSO in patients who are psychologically unfit to tolerate it [10], although we have observed that patients are generally able to withstand the treatment without particular difficulties, probably due to the resilience generated by the condition itself. As far as contractures and infections are concerned, the former may be resolved by a course of aggressive physical therapy, while the latter should be prevented by meticulous pin site hygiene [5].

Several proposals have been put forward to reduce the duration of external fixation and limit its drawbacks. Some authors strive to avoid the cumbersomeness of a proximal ring fixator by resorting to a combination of a monolateral and a circular fixator [24]. Others employ exclusively monolateral fixators. In some reports, the proximal osteotomy is stabilized by means of plates, reserving the fixator for the lengthening procedure [28]. Finally, some publications only use internal fixation devices, the lengthening of the limb being achieved by motorized nails [33]. Although satisfactory results have been obtained with all the systems above, our experience is limited to the use of (monolateral and circular) external fixators, having found no differences between the two options in terms of outcomes or complication rates.

The main criticism leveled against PSO is related to the alterations caused to the proximal femoral anatomy, which could complicate the performance of a THA at a later stage [15,22]. Nevertheless, some authors have demonstrated the feasibility of fitting a prosthesis into the hip joint following a reconstruction procedure [20,34]. Furthermore, it is our belief that the benefits provided by PSO over the years largely outweigh the technical difficulties inherent in a (potential) subsequent joint replacement. In addition, as mentioned above, the failure rate associated with PSO is very low and its positive effects have been shown to be considerably long-lasting, which makes it unlikely that a large number of patients will require conversion to a THA [5,20].

This study is not without limitations. First and foremost, it is an observational study with a small sample size. This small sample size can be explained by the (fortunately) reduced number of patients who require a POS, which means that all the reports on the technique are inevitably faced with the same difficulty. This limitation, together with the multiplicity of conditions that could give rise to a chronically unstable hip, makes it difficult to compare across different series and precludes the performance of a randomized clinical trial to isolate the factor to be analyzed. However, we believe that the analysis presented here is significant from a clinical perspective as it combines the presentation of the results of a series of patients treated in the pediatric orthopedics unit of a hospital in an industrialized country with the most extensive literature review conducted to date on the subject. It should also be mentioned that patient satisfaction was not evaluated with a validated questionnaire, although all subjects claimed to be satisfied with the result obtained.

Severe hip damage takes on different forms depending on the condition that causes it and the patients that suffer its consequences. Despite the existence of multiple treatment options, the most effective way to manage the condition is still a matter of controversy. Each option is associated with its pros and cons, but it should be considered that decisions are often influenced by the surgeon’s age, attitudes and set of values. Our in-depth review of the published literature made us conclude that pelvic reconstruction using PSO is an alternative capable of improving gait performance, relieving pain, reducing associated spine deformities and eliminating leg length discrepancies in children and young adults with severe hip instability. Moreover, the technique offers a durable solution that does not necessarily preclude a subsequent revision THA [5,20,34].

## 5. Conclusions

Pelvic support osteotomy combined with distal femoral lengthening is a valuable salvage option for managing severely damaged hips in children and adolescents. In our series, it provided satisfactory clinical results, and complications were mild and manageable.

## Figures and Tables

**Table 1:** Anthropometric data, etiology and associated deformities.

Case	Sex	Age	Side	Etiology	Necrosis	Dislocation	Stiffness	Coxa Vara	Coxa Valga	Genu Valgum
1	Male	16	Right	Slipped capital femoral epiphysis	Yes	-	Yes	-	-	-
2	Male	10	Right	(Non-tuberculous) septic arthritis	Yes	-	-	Yes	-	-
3	Male	19	Right	Spinal tumor-derived sciatic nerve palsy	Yes	-	Yes	-	-	-
4	Female	10	Left	Arthrogryposis	-	Yes	-	-	Yes	Yes
5	Male	16	Right	Developmental dysplasia of the hip	Yes	-	Yes	-	-	-
6	Male	12	Right	Slipped capital femoral epiphysis	Yes	-	Yes	-	-	-
7	Male	10	Right	(Tuberculous) septic arthritis	Yes	-	Yes	-	-	-
8	Female	9	Left	(Non-tuberculous) septic arthritis	Yes	-	Yes	-	-	-
9	Female	14	Left	Congenital short femur	-	Yes	-	-	-	-
10	Male	16	Right	Congenital short femur	-	Yes	-	-	-	-
11	Female	12	Right	(Non-tuberculous) septic arthritis	Yes	-	Yes	-	-	-
12	Male	12	Right	Developmental dysplasia of the hip	Yes	-	Yes	-	-	-
Total	8M/4F	13	9R/3L	-	9	3	8	1	1	1

**Table 2:** Leg length discrepancy and ambulation.

Case	PreOp Leg Length Discrepancy (cm)	Lengthening (cm)	FollowUp Leg Length Discrepancy (cm)	ExFix Time (days)	ExFix Index (days/cm)	PreOp Trendelenburg	FollowUp Trendelenburg
1	8	8	1	270	33.8	Severe (+++)	Mild (+)
2	7	7	2	210	30.0	Severe (+++)	Negative (−)
3	10	10	0	360	36.0	Severe (+++)	Mild (+)
4	10	10	2	320	32.0	Severe (+++)	Moderate (++)
5	5	5	0	180	36.0	Severe (+++)	Negative (−)
6	7	7	2	210	30.0	Severe (+++)	Mild (+)
7	10	10	0	330	33.0	Severe (+++)	Mild (+)
8	9	9	3	225	25.0	Severe (+++)	Mild (+)
9	9	9	0	330	36.7	Severe (+++)	Mild (+)
10	7	7	1	240	34.3	Severe (+++)	Moderate (++)
11	6	7	0	210	30.0	Severe (+++)	Negative (−)
12	8	8	0	270	33.8	Severe (+++)	Mild (+)
Total	8	8.1	0.9	263	32.6	12 +++	2 ++; 7 +; 3 −

**Table 3:** Range of motion progression.

Movement	Stage	Degrees	*p*-Value
Flexion	Preoperative	96.7	0.002
	Follow-up	128	
Extension	Preoperative	7.5	0.006
	Follow-up	24.2	
External rotation	Preoperative	10	0.003
	Follow-up	38.3	
Internal rotation	Preoperative	9.2	0.004
	Follow-up	24.2	
Abduction	Preoperative	18.3	0.002
	Follow-up	37.5	
Adduction	Preoperative	12.5	0.003
	Follow-up	22.5	

**Table 4:** Complications.

Case	Delayed Healing	Fracture Regenerate	Pin tract Infection	Joint Contracture	Vascular Injury	Problems	Obstacles	Complications
1	-	-	-	Yes	-	0	0	1
2	-	Yes	-	-	-	1	0	0
3	Yes	Yes	-	-	-	2	0	0
4	Yes	-	Yes	-	-	1	1	0
5	-	-	-	-	-	0	0	0
6	-	-	-	-	-	0	0	0
7	-	-	-	-	-	0	0	0
8	-	-	-	-	-	0	0	0
9	-	-	Yes	-	-	1	0	0
10	-	-	-	Yes	-	0	0	1
11	-	-	-	-	Yes	1	0	0
12	-	-	Yes	-	-	1	0	0
Total	2	2	3	2	1	7	1	2

**Table 5:** Other studies.

Study	N	Sex	Age (Mean)	Follow-Up (Months)	PostOp Leg Length Discrepancy (cm)	ExFix Time (Months)	ExFix Index (Months/cm)	Lengthening (cm)	PostOp Trendelenburg (%)
Umer (2014) [5]	37	18 M/19F	23.3	-	1.0	-	-	-	-
El Mowafi (2005) [4]	25	8M/17F	22.4	54.0	-	7.0	1.4	5.0	20%
Marimuthu (2011) [16]	12	7M/5F	23.0	59.4	0.9	7.3	-	-	25%
Kocaoglu (2002) [7]	14	2M/12F	20.0	68.0	-	7.0	1.6	4.4	21%
El Rosasy (2014) [15]	16	9M/7F	23.0	85.6	0.0	4.6	1.6	2.8	23%
Inan (2005) [19]	11	0M/11F	25.2	36.0	-	-	-	-	83%
Rozbruch (2005) [13]	8	-	11.2	60.0	0.8	4.7	0.8	5.7	25%
Mahran (2011) [18]	20	5M/15F	21.5	6.0	1.1	6.4	-	-	45%
Schiltenwolf (1996) [20]	24	-	17.0	204.0	-	-	-	-	50%
Inan (2005) [21]	16	2M/14F	25.3	52.0	1.0	7.1	-	-	25%
Masquijo (2008) [22]	13	7M/6F	13.7	36.4	1.3	7.5	-	-	54%
Gursu (2011) [23]	20	-	22.6	33.5	1.6	12.0	1.9	6.3	67%
Luo (2020) [24]	17	5M/12F	20.6	64.3	-	-	-	-	0%
Ghanghurde (2017) [25]	6	5M/1F	10.0	48.0	1.0	6.0	-	-	0%
Wu (2019) [26]	13	2M/11F	24.2	31.2	1.5	-	-	-	0%

## Data Availability

The data presented in this study are available on request from the corresponding author due to its medical nature.

## Abbreviations

The following abbreviations are used in this manuscript:

THA	Total hip arthroplasty
PSO	Pelvic support osteotomy
